# Generating genius: how an Alzheimer’s drug became considered a ‘cognitive enhancer’ for healthy individuals

**DOI:** 10.1186/1472-6939-15-37

**Published:** 2014-05-12

**Authors:** Lucie Wade, Cynthia Forlini, Eric Racine

**Affiliations:** 1 Neuroethics Research Unit, Institut de recherches cliniques de Montréal, 110 avenue des Pins Ouest, Montréal, QC H2W lR7, Canada; 2 Department of Medicine and Department of Social and Preventive Medicine, Université de Montréal, 2900 Boulevard Edouard-Montpetit, Montréal, QC H3T 1J4, Canada; 3 Departments of Neurology and Neurosurgery, 3801 University Street, Montreal, QC H3A 2B4, Canada; 4 Division of experimental Medicine and Biomedical Ethics Unit, 1110 Avenue des Pins Ouest, Montreal, QC H3A 1A3, Canada; 5 Ethics Program Office, Jewish General Hospital, 3755 Cote-St-Catherine Road, Montreal, QC QC H3T 1E2, Canada; 6 The University of Queensland, UQ Centre for Clinical Research, 3755 Cote-St-Catherine Road, Herston, QLD 4029, Australia

## Abstract

**Background:**

Donepezil, an acetylcholinesterase inhibitor used in the treatment of Alzheimer’s disease, has been widely cited in media and bioethics literature on cognitive enhancement (CE) as having the potential to improve the cognitive ability of healthy individuals. In both literatures, this claim has been repeatedly supported by the results of a small study published by Yesavage *et al.* in 2002 on non-demented pilots (30–70 years old). The factors contributing to this specific interpretation of this study’s results are unclear.

**Methods:**

We examined print media and interdisciplinary bioethics coverage of this small study, aiming to provide insight into how evidence from research may be shaped within different discourses, potentially influencing important policy, ethics, and clinical decisions. Systematic qualitative content analysis was used to examine how this study was reported in 27 media and 22 bioethics articles. Articles were analyzed for content related to: (1) headlines and titles; (2) colloquialisms; and, (3) accuracy of reporting of the characteristics and results of the study.

**Results:**

In media and bioethics articles referencing this small study, strong claims were made about donepezil as a CE drug. The majority of headlines, titles, and colloquialisms used enhancement language and the majority of these suggest that donepezil could be used to enhance intellectual ability. Further, both literatures moved between reporting the results of the primary study and magnifying the perceived connection between these results and the CE debate that was alluded to in the primary study. Specific descriptions of the results overwhelmingly reported an improvement in performance on a flight simulator, while more general statements claimed donepezil enhanced cognitive performance. Further, a high level of reporting accuracy was found regarding study characteristics of the original study, but variable levels of accuracy surrounded the presentation of complex characteristics (i.e., methods) or contentious properties of the CE debate (i.e., initial health status of the study subjects).

**Conclusions:**

Hyped claims of CE effects cannot be completely accounted for by sheer inaccuracy in reporting. A complex interaction between the primary and secondary literature, and expectations and social pressures related to CE appears to drive enthusiastic reports.

## Background

The phenomenon of cognitive enhancement (CE)—the idea that one might improve upon their typical, or “healthy,” level of cognitive performance through the use of pharmaceuticals originally developed to treat medical conditions—has garnered media attention [1,2] and generated scholarly debate. Within both literatures, arguments have been made in favour of providing these so-called “CE drugs”^a^ to professionals, such as surgeons and pilots, and school children [3-6]. Such arguments contribute to the perception that CE drugs are both efficacious and in demand, which has fuelled calls for the development of regulatory frameworks to control their use [7]. Already, medical professional societies such as the American Academy of Neurology [8,9] and governmental ethics committees [10] have deliberated over the proper policy response to the use of pharmaceuticals for enhancement purposes^b^. Yet, an informed public, medical, and ethical response to CE remains limited by a dearth of evidence supporting the possibility of such a practice. Indeed, studies examining the body of evidence informing this debate have found it to be inconsistent [11], inadequate and insufficient [12], and study outcomes to be incompatible and limited in number [13], making any generalizable conclusions elusive.

Why CE has generated a high level of public and professional attention despite a limited body of evidence is a provocative question. Previous research has shown that the media may play a role in disseminating information, driving interest and fuelling misrepresentation of the level of evidence [14-16]. However, it is increasingly acknowledged that the media does not act in isolation [17]. Overstatements of therapeutic effect in media articles have been found to be “faithfully reported” [18] from the conclusions of scientific articles. The media may be overly deferential to claims made with the authority of science, which can be problematic as the claims referenced may involve speculation [19]. Further, a “spatial dynamic,” [20] may exist whereby expectations of positive outcomes are generated from little evidence due to the interaction of multiple stakeholders, including research communities, funding agencies, and patient groups as well as the media. Such a multimodal interaction may account for the interest in one pharmaceutical, methylphenidate, as a CE drug. Specifically, speculation over the ability of methylphenidate to change lives may have introduced “a cycle of expectation” [13]. This expectation—like the speculation it arises from—has loose connections with empirical evidence, making it resistant to detailed discussions of the limitations of current efficacy data that should curtail interest [13].

### Donepezil as a case example to explore the generation and persistence of academic and media claims of CE effects despite limited evidence

The study of donepezil provides a possible window into how CE more broadly became and remains a topic of focused interest for the public and academia alike, despite a limited body of supporting evidence. The evolution of donepezil, an acetylcholinesterase inhibitor, from a treatment for Alzheimer’s disease (AD) to a contender in the CE debate is intriguing. First, the medical value of donepezil in AD has been contested. It took funding agencies, such as the UK’s NICE, decades to gather sufficient evidence of effect to support their endorsement of the drug as a treatment for mild and moderate dementia [21]. Further, in this medical context donepezil has been described as a “cognitive enhancer” because it slows down AD-related cognitive decline. However, this use of the term CE does not refer to the phenomenon of CE that we are discussing in this paper (see note b, above), creating an opportunity for misinterpretation of the drug’s effects and applications if studies on CE in a clinical population are confounded with studies of CE which specify a “healthy” population. Indeed, out of 446 studies on the effect of acetylcholinesterase inhibitors reviewed by Repantis et al., only 20 had results relevant to the cognitive enhancement of already *healthy* individuals [12].

Second, media and bioethics articles that claim donepezil has a CE effect rely heavily on the results of a single study, which in addition to being a very small study (n = 18) with limited results, is open to different interpretations in the way described above: references to CE may refer to the phenomenon of enhancing healthy individuals, or could connote the type of CE referred to in the AD literature). The study, published in 2002 in *Neurology*, reported an effect of donepezil on the ability of non-demented pilots to retain complex skills in a flight simulator [22]. Specifically, the study (which we will abbreviate as the “Donepezil flight simulator study” or DFSS^c^ in this paper, see Table 1) found that “[d]onepezil appears to have beneficial effects on retention of training on complex aviation tasks in nondemented older adults” [22]. The American Academy of Neurology, like many others, cited this study as supporting evidence for the existence of CE. However, the use of the results of this study as evidence that donepezil is a cognitive enhancer is problematic for several reasons. First, the authors of the DFSS have clarified that the applicability of their data is limited, stating: “[o]ur results should not be interpreted as a recommendation for the use of donepezil as a drug to improve flight performance” [23]. Specifically, their results are based on a simulated flight experience, not the piloting of an airplane.

**Table 1:** Brief overview of the Donepezil flight simulator study (DFSS)

**Background and methods**	In 2002, Yesavage *et al*. conducted a study on 18 pilots, aged 30–70 with a mean age of 52, who were split into placebo and control groups. The groups were randomized, and after seven 75 minute long practice tests on a flight simulator (where the baseline for the study was also calculated), the drug group was administered 5 mg of donepezil per day for 30 days. On day 30 both groups performed two more flight simulator tests. The primary outcome measure of the study was the change in flight score from the flights performed on day 30, when compared with those on day zero. Four different flight components were assessed during the flight simulations: communication, traffic avoidance, emergencies, and approach to landing.
**Results and conclusions**	The results of the study state: “flight performance of the pilots in the donepezil group changed little from performance after the initial training to 30-day post-treatment… whereas it declined in pilots in the placebo group” (see Table 3 for a more detailed description of the results).

Second, in the introduction section of the DFSS, the authors justify their use of donepezil as the intervention drug based on previous work that has led to the “‘cholinergic hypothesis’ which proposes that part of age-related cognitive decline is caused by reduced cerebral cholinergic function” [22]. One of the goals of the study was to test whether donepezil could improve flight performance in older pilots as the United States prevents pilots over 60 from flying due to concerns about the effects of aging [22] on flying ability. Thus, one of the premises of this study was the idea that donepezil may improve the performance of pilots whose cognitive and psychomotor skills may have been *affected by aging*. Though the idea that pilots’ ability is affected by aging is a controversial claim, the authors of the DFSS justified donepezil as the appropriate intervention on these grounds. Thus, it appears the study set out to determine whether donepezil could improve performance in the flight simulator by treating the reduction in cholinergic function. Interpreted in this way, the study may be considered as an investigation of a treatment for age-related cognitive decline (which would connote a disease state), rather than as an enhancement, casting doubt on the generalizability of the study’s results to the CE debate.

Third, subsequent detailed reviews of studies on the effects of acetylcholinesterase inhibitors on healthy populations, which included the DFSS, have since concluded that none of the studies provide sufficient evidence to support the CE claims made about donepezil [12,24]. Finally, in contrast to the non-medical use of stimulants for CE [25,26], no strong prevalence data exists that bolsters claims of a CE effect by providing evidence of a perceived CE effect by users. However, donepezil, supported by reference to the DFSS, persists as a key reference in the CE debate, contributing to the perception that CE is possible, and, as already seen, shaping discussions about clinical practice.

Evidently there is tension between, on the one hand, academic and media discourse about donepezil as a CE drug and, on the other, the level of evidence supporting this claim. This study aimed to examine how media coverage and bioethics discussion of the DFSS has shaped donepezil as a CE agent, evaluating what factors might account for the presentation of an interpretation of the DFSS in popular and scholarly articles that is not promoted by the study’s authors. Additionally, we sought to explore the ethical issues at stake—specifically, the impact of hyped claims about the CE on autonomous decision-making and the physician-patient relationship—as CE continues to be propagated as a possibility.

## Methods

This study analyzes the reporting of one study, the DFSS, in public and academic literature. We conducted a systematic review of international (i.e., including Canada, US, UK) print media (M) and interdisciplinary bioethics literature (BE) where references to the DFSS appeared within a discussion of CE.

### Sampling

To develop our media sample we searched the *Factiva* database using key word searches to find English language media articles on CE published between 2002 and 2009, inclusively, based on existing media sampling methods [15,27]. The start date was selected to capture media articles released following the publication of the DFSS. Key word searches were used to broadly identify articles that addressed cognitive enhancement, donepezil, and the DFSS. The terms “donepezil”, “Aricept”, “E2020”, “acetylcholinesterase”, “enhance”, “Yesavage”, “Mumenthaler”, “*Neurology*”, “pilots”, “flight simulator”, “memory”, “illicit drugs”, “study aid,” and their variants (e.g., singular and plural forms) were used in different search combinations to maximize relevant results. Initial searches yielded 339 potential articles. To develop our bioethics sample we performed citation searches in the academic databases *Pubmed, Google Scholar, Medline*, and *Proquest.* A total of 154 articles (7, 85, 21, 41, respectively) were identified that specifically referenced the DFSS.

Articles were excluded from our samples if they were duplicates, referenced a different study by the same authors, focused on the medical use of donepezil (e.g., Alzheimer’s, schizophrenia, bipolar, mild cognitive impairment, impaired attention, dementia), or referred to the study in the wider context of animal research. For the bioethics sample, non-peer reviewed and non-English language publications, as well as articles published after 2009 were also excluded. Inclusion criteria were then applied, restricting our samples to only include articles that discussed CE. The media sample was further examined to ensure that each article either made direct reference to the DFSS or made indirect reference to the study by presenting at least four study characteristics (e.g., year, journal, author, author affiliation, subjects, methods, results, limitations, ethical issues raised) that clearly established its identity. Our final sample included 27 international print media articles and 22 interdisciplinary bioethics papers.

### Content and discourse analysis

A coding guide was developed for the systematic analysis of articles. It was inductively generated and informed by previous qualitative content analysis of media coverage of CE performed by two of the authors (CF and ER; [2]). Extensive pretesting on a sub-set of articles was conducted to tailor this previous coding guide to the specific research objectives of this project. The coding strategy was “rich” and not mutually exclusive. Systematic analysis of content captured information related to three distinct categories within each article: (1) headlines and titles; (2) colloquialisms used to refer to donepezil; and, (3) reporting of the characteristics and results of the DFSS. One author (LW) was responsible for conducting the initial coding. One co-author (CF or ER) reviewed the coding and the other (ER or CF) resolved any disagreements over challenging codes. Complex coding was conducted using the QSR NVivo 8 software (Doncaster, Australia) while simpler components of coding were carried out in Excel spreadsheets.

### Analysis of headlines and titles

Headlines and titles were separated from the body of the articles and coded based on 1) whether they claimed an enhancing property; 2) what property, if any, was highlighted; and, 3) how the context of CE was portrayed (i.e., if they suggest that CE is currently happening, is anticipated, is questionable, or is undesired).

### Analysis of colloquialisms

Colloquialisms, such as “smart pill” [28], which were used to explicitly refer to donepezil, were coded separately. To ensure connection with the DFSS, colloquialisms were coded only if they were found within the paragraph where the DFSS was referenced; or, in bioethics articles, if there was a direct structural connection to the reference (i.e., the colloquialism was found in the heading of the section where the study was referenced). Colloquialisms were coded with respect to the enhancement property they imply (e.g., CE in general or memory specifically).

### Analysis of result claims, qualifying clauses, and study characteristics

Media and bioethics articles were systematically coded to capture how they conveyed results, qualifying clauses and study characteristics. Only information that was clearly linked to the reference of the DFSS found in each bioethics and media article was retained for coding. The specific coding guide applied to the reporting of the DFSS included the identification of: (1) the characteristics of the study (e.g., sample size, subject information, tests used, dosage information); (2) statements of the results or findings of the study broken into two levels: “specific” results (i.e., an explicit statement of the results of the study) and “extended” results (i.e., when the author of the article interpreted or restated the “specific” results to introduce the study, or to connect it to wider issues); and, (3) qualifying clauses related to the rigor or limitations of the study. The original study was also coded using the coding guide used for both media and bioethics articles. Descriptive statistics were used to quantify and qualify the distribution of headlines, titles, colloquialisms, and claims made about the results of the DFSS within the different codes, as well as qualifying clauses.

### Specific analysis of study characteristics by Euler’s circles

**Figure 1:**
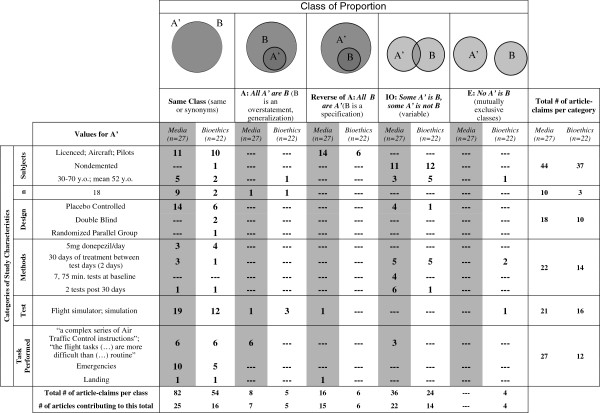
**Accuracy of study characteristics reported.** The first column gives values for “**A**’”, the claims made in the study, while the following five columns provide the number of articles that made “**B**”, alternative claims that describe the same study characteristic, in each of five Euler classes (representing varying degrees of accuracy). Absolute numbers represent how many different articles had at least one B description in each sub-category. Each article was counted only once per sub-category (e.g., non-demented, placebo controlled) of study characteristic even if it made two or more separate claims that fell into that sub-category. This metric is captured by the term “article-claim”. Thus, the numbers in each cell refers to the number of “article-claims” made per sub-category added together for each category in each class. To calculate the total number of article-claims made per class, the numbers of article-claims per category were added together. Theoretically, a single article could account for 15 category claims since they could have made a claim about each sub-category of study characteristics. To calculate the total number of article-claims made per category, the numbers of article-claims per class were added together. Here, a single article could account for a maximum of 5 times the number of sub-category claims that is possible in each category, since they could have made a claim about each sub-category that fell into each class.

Logical analysis by Euler’s circles was applied to reports of the characteristics of the DFSS found in media and bioethics articles to examine how accurately they reflect those of the study itself. Euler’s circles provide a clearly visible and easily replicated method for the logical analysis of how closely a claim relates to or represents another, primary, claim [29]. Logical analysis was conducted by division of secondary claims into five distinct logical classes, called proposition classes, each representing a different relationship between the primary and secondary claims (see Figure 1 for proposition classes) [29,30].Media and bioethics reports of study characteristics were identified as B (claims; B) and actual study characteristics as A’. Claims made by media and bioethics articles were then placed in the proposition class that best reflected their relationship to the A’ characteristic. The distribution of claims between proposition classes and categories of study characteristics was then calculated on a one article per category basis (even if that article made two distinct claims), yielding a basic unit of analysis we refer to as an “article-claim” (see the legend of Figure 1 for further detail).

## Results

### Headlines and titles

To assess the main themes conveyed to readers of articles that cite the DFSS, we analyzed the media headlines and bioethics titles (see Additional file 1 and Additional file 2 for complete lists of articles). The most common topic portrayed by media headlines was a general claim of an enhancement effect (N = 17/27; 63%), which was almost always related to the use of a pharmaceutical (N = 16/17; 94%). The majority of these headlines related the enhancement effect to intellect or cognition (N = 9/17; 53%; e.g., *Brain-boosting drugs could soon become the smart choice*[31]) or stated that an enhancement effect was currently possible (N = 13/17; 76%) or anticipated (N = 2/17; 12%). A minority of headlines presented wary or skeptical comments regarding the possibility of enhancement (N = 6/17; 35%; e.g., *Can taking a pill make you brainy?*[32]), and no headlines implied that CE is undesirable. Within the headlines that did not focus on intellect or cognition, enhancement effects on general mind/brain (N = 3/17; 18%), memory (N = 3/17; 18%), attention (N = 2/17; 12%), youth (N = 1/17; 1%), or performance (N = 1/17; 1%) were conveyed.

Of the nine headlines that did not present an enhancement effect, the majority claimed that there had been a breakthrough for memory problems (N = 7/10; 70%; e.g. *Memory drug has landed*[33]), and the majority of these headlines specified that the breakthrough was pharmacological (N = 4/7; 57%).

Most titles of the bioethics articles (N = 16/22; 73%), featured the term “enhancement;” the majority specifically referring to “neurocognitive enhancement” (N = 11/16; 69%). Bioethics titles generally suggested a more philosophical or critical approach to the issue of CE than media headlines did, but they still implied that CE was a possibility as they introduced the potential for concern, (N = 10/16; 63%; e.g., *Neurocognitive enhancement: what can we do and what should we do?*[34]). In contrast to the media headlines, only 19% (N = 3/16) of bioethics titles related enhancement to pharmaceutical use.

Additional themes in the bioethics titles were: 1) the role of medicine with respect to CE (N = 8/22; 36%); mainly, the implications of the sliding treatment-enhancement scale, though two articles focused on the possibility of cognitive enhancement for physicians; and, 2) the progress or evolution of CE and appropriate responses for the future (N = 6/22; 27%). None of the titles in our sample implied medical use of pharmaceuticals.

### Colloquialisms

To determine whether explicit connections were made between donepezil and CE, we examined the colloquialisms that were used to refer to donepezil. Colloquialisms were used in 52% of media articles (N = 14/27) and 45% of bioethics articles (N = 10/22; Table 2; [24,28,31,33-54]) to refer to donepezil, with a total of 20 distinct colloquialisms used in media and 12 in bioethics articles. The majority of colloquialisms used enhancement language (N = 10/20 M, 50%; N = 11/12 BE, 92%). A large proportion of both media and bioethics colloquialisms that used enhancement language specifically referred to cognitive or brain enhancement (N = 4/10 M, 40%; N = 8/12 BE, 73%, e.g., “brain-enhancing drugs” [35,36]). Additionally, many colloquialisms referenced an effect on intelligence though they did not use explicit enhancement language. When these intelligence colloquialisms were combined with those that referred explicitly to cognitive enhancement, 50% of media and 75% bioethics colloquialisms conveyed an effect on intelligence (N = 10/20 M, N = 9/12 BE).

**Table 2:** Colloquialisms* used in media and bioethics discourses that explicitly refer to the drug donepezil**

	**Media**	**Bioethics**
Cognitive/brain enhancement	“the new ‘cognitive enhancers’” [37]	“cognitive enhancement drugs” [38]
	“brain-enhancing drugs” [36]	“enhancers of cognitive performance” [39]
	“brain-enhancing drugs” [35]	“cognition enhancers” [40,41]
	“mind-enhancing medicine” [37]	“brain-enhancement drugs” [42]
		“neuroenhancement drugs” [43]
		“psychological enhancements” [44]
		“‘magic potions’ to enhance our ‘wisdom’” [45]
“Smarts”	“so-called ‘smart drugs’” [46]	
	“so-called smart pills” [31]	
	“smart pill” [28]	“smart drugs” [45]
	“the ‘older’ smart drugs” [33]	
	“the existing smart drugs” [33]	
	“cognition drugs” [47]	
Memory enhancement	“memory-enhancing drugs” [48]	“memory enhancing agents” [45]
	“memory-enhancing pills” [28]	
	“Alzheimer drug boost for healthy memory” [49]	
	“memory-enhancing medications” [50]	
Others	“performance-enhancing drugs” [48]	“currently available enhancers” [24]
	“the safety-enhancing drug” [37]	“enhancements” [54]
	“these ‘fountain of youth’ drugs” [48]	
	“new brain boosters” [51]	
	“brain cosmetics” [52]	
	“a memory pill” [53]	

*Terms are divided based on the type of effect implied (e.g., brain enhancement, memory enhancement).

**Or group of drugs containing donepezil.

Enhancement colloquialisms also specified an effect on memory (N = 5/10 M, 50%; N = 1/12 BE, 1%) e.g., “memory enhancing pills” [28]), youth (N = 1/10 M,), safety (N = 1/10 M) and performance (N = 1/10 M). Two bioethics articles claimed the general effect of enhancement, without specifying what characteristic or properties are enhanced (e.g., “enhancements” [54]). Only one media article used a colloquialism that could refer to a medical treatment, rather than enhancement (e.g., “a memory pill” [53]).

### Reporting of the results of the DFSS

To determine how the results of the DFSS were reported in media and bioethics discourses, we summarized the claims made in the primary study (Table 3), as well as those found in media [28,31-33,35,36,46-53,55-66] and bioethics articles [6,8,24,38-45,54,67-75] (Additional file 3 and Additional file 4).

**Table 3:** Statements of the results of the DFSS as they appeared in the original paper

	**Findings of the DFSS**
**Abstract**	“After 30 days of treatment, the donepezil group showed greater ability to retain the capacity to perform a set of complex simulator tasks than the placebo group, p 0.05.”
	“Donepezil appears to have beneficial effects on retention of training on complex aviation tasks in nondemented older adults.”
**Results**	“After 30 days of treatment, there was a significant difference between the donepezil group (n = 9, mean age 51.2 years) and the placebo group (n = 9, mean age 53.1 years) in flight performance change (F 6.1, p 0.05, effect size 0.58)”
	“Overall, flight performance of the pilots in the donepezil group changed little from performance after initial training to 30-day post-treatment (0.06 z-score units; SD 0.31), whereas it declined in pilots in the placebo group (0.24 z-score units; SD 0.19)”
	“To help focus the discussion of the likely locus of drug effects, post hoc analyses of flight component difference scores were computed. These scores reflect differences in performance between treatments over the course of treatment. Examination of the figure suggests the largest effects of donepezil were on the emergency scanning (effect size 0.56) and the approach to landing scores (effect size 0.52)”
**Discussion**	“Given the extensive literature on the effects of acetylcholinesterase inhibitors on memory, we were not surprised to find some effects of the drug on ability to retain a practiced skill in pilots”
	“Nonetheless, these results are consistent with previous studies in nondemented adults that have reported that cholinesterase inhibitors improve cognitive performance.”
	“The association of cholinergic drugs with better attention has lead investigators to suggest that part of the benefit of cholinergic drugs on memory performance may be mediated through attentional components involved in working memory. This suggestion is supported by the current data that show the strongest drug effects on emergency tasks and the approach to landing. The emergency tasks involve visually scanning the instrument panel for aberrant readings. The approach to landing requires sustained divided attention to maintain proper altitude, speed, and heading”

### The DFSS

In its abstract, the DFSS claimed that donepezil had “beneficial effects on retention” as pilots who took donepezil “showed greater ability to retain the capacity to perform” (Table 3). In the results section, a significant difference was described between the drug and control groups related to “in flight performance change.” Here the authors clarified that “flight performance of the pilots in the donepezil group changed little, whereas it declined in pilots in the placebo group” (Table 3). In the discussion the authors stated that their results were consistent with those of previous studies that “reported that cholinesterase inhibitors improve cognitive performance” (Table 3). They also extrapolated a connection between cholinesterase inhibitors, working memory and memory performance (Table 3).

### Media and bioethics articles

All media and bioethics reports of the results of the DFSS, save one bioethics article, enthusiastically portrayed a beneficial effect of donepezil. This lone bioethics critique occurred at the level of *extended* results where the article expressed concern that: “the available evidence does not appear to support the widely cited conclusion that donepezil improves the retention of training” [24].

### Specific results

Reporting of *specific* results was high (N = 23/27 M, 85%; N = 18/22 BE, 82%; Additional file 3 and Additional file 4), yielding a total of 36 M and 19 BE specific results claims. Improvement language was used in 94% of media claims (N = 34/36 M) and 84% of bioethics claims (N = 16/19B). Of those claims that used improvement language, improved task performance by the pilots who took donepezil was mentioned in 71% of media and 63% of bioethics claims (N = 24/34 M; N = 10/16 BE), mainly referring to their performance in the flight simulator and emergencies; memory performance was mentioned in 29% of media claims and 25% of bioethics claims (N = 10/34 M; N = 4/16 BE); and brain performance was mentioned in only 6% of bioethics claims (N = 1/16 BE).

Explicit enhancement language (i.e., the term enhancement or a variation on that term) was used in 3% of media claims and 11% of bioethics claims (N = 1/36; N = 2/19). The media claim that used enhancement language referenced brain performance, while both bioethics claims referenced task performance.

### Extended results

The majority of articles also reported *extended* results (N = 16/27 M, 59%; N = 13/22 BE, 59%; Additional file 3 and Additional file 4), yielding a total of 23 M and 14 BE extended results claims. Improvement language was used in 65% of media claims (N = 15/23 M) and 36% of bioethics claims (N = 5/14 BE). Of those claims that used improvement language, improved task performance by the pilots who took donepezil was mentioned in 33% of media and 20% of bioethics claims (N = 5/15 M; N = 1/5 BE); memory performance in 33% of media claims and 40% of bioethics claims (N = 5/15 M; N = 2/5 BE); and brain performance in 33% of media claims and 40% of bioethics claims (N = 5/15 M; 2/5 BE).

Explicit enhancement language was used in 30% of media claims and 50% of bioethics claims (N = 7/23 M; N = 7/14 BE). Enhanced memory was referenced in 57% of media claims and 43% of bioethics claims (N = 4/7 M; N = 3/7 BE); brain performance was referenced in 43% of media claims and 57% of bioethics claims (N = 3/7 M; N = 4/7 BE).

### Qualifying clauses

We also examined the qualifying clauses made in the DFSS and reported by a handful of media and one bioethics article (N = 4/27 M, 15%; N = 1/22 BE, 5%). The authors of the original study presented both practical and epistemic qualifications. *Practical* qualifications warned of potential side-effects of the drug (in the CE context) and a need for a larger sample size or further tests. These qualifications were each only reported by two media articles (7%). The *epistemic* qualification that “these results should not be interpreted to advocate widespread use of donepezil in nondemented populations” [22]) was reflected in three media articles (11%). The second epistemic qualification that “[a]lthough these findings may support interpretations of the effects of cholinergic augmentation on cognitive processing, the precise neurochemical mechanisms of action remain to be fully delineated” [22] was only reported by one bioethics article (5%).

### Reporting of the study characteristics of the DFSS

**Figure 2:**
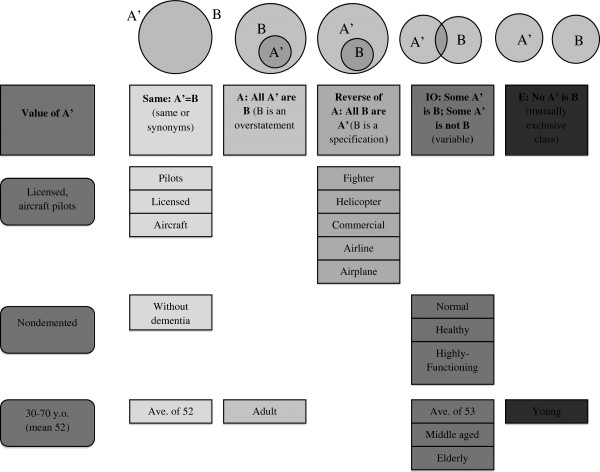
**A visualization of the characteristics of the “subjects” category (from Figure**1**) as stated in the DFSS (A’) and their translation into media and bioethics article-claims (B).** This study category represented the largest total number of article-claims and its article-claims were distributed across the various classes. The figure shows how claims can represent the same characteristic category but fall into different proposition classes. This figure also demonstrates the range of qualitative differences possible in the translation of discrete categories of information.

To establish whether the characteristics of the DFSS (e.g., subject information, sample size (n), study design) were accurately reported in media and bioethics literatures, we directly compared the claims of the primary study to those made in media and bioethics articles (Figure 1). All media and all but one bioethics article reported at least one study characteristic. Figure 1 shows the numeric breakdown of media and bioethics article-claims as they correspond to each category and are distributed across the Euler classes. The majority of article-claims made were synonymous (i.e., accurate) with those found in the DFSS (“same” class; Figure 1).However, when the categories of study characteristics were analyzed independently, we found two categories that did not reflect this trend: the “subjects” category and the “methods” category. The “subjects” category was also the category that had the largest total number of article-claims (Figure 1). In addition to finding a high number of article-claims about the “subjects” category in the “same” class, we found a high number in class “IO,” the “Some A’ are B, some A’ are not B” class. In this class, part of the claim might be accurate, or under certain circumstances it may be accurate, but not always. We also found a high number of article-claims in the specifications (“All B are A’”) class (Figure 1). Figure 2 demonstrates the range of qualitative differences possible in the translation of discrete categories of information with respect to information about study subjects. As the category with the largest total number of article-claims this category is an illustrative example of how claims can represent the same characteristic category but fall into different proposition classes.

## Discussion

This study examined media and bioethics coverage of the DFSS, which serves as supporting evidence in the CE debate. We specifically focused on how the DFSS is reported in media and bioethics articles that engage in the debate in order to characterize how secondary literature has contributed to the claim that donepezil has CE effects.

Our key findings demonstrate that both media and bioethics articles use the DFSS as support to make strong claims about the use of donepezil for CE purposes, specifically for increasing intelligence and memory. These claims are most pronounced in the headlines and titles of articles that engage in the CE debate, as well as in the colloquialisms that are used to explicitly refer to donepezil within the context of a discussion about CE (Table 2). Emphasis is placed on the possibility that donepezil could affect cognition or intellectual capacity while consistent use of enhancement language implies that this effect is both above one’s “healthy” level of cognition and is a desirable or laudable goal. This supports the previous findings of a study that found that media and bioethics discourses were generally enthusiastic in portraying CE effects of methylphenidate on cognition [2]. However, we did see a difference between headline and title references to pharmaceutical use as well as to the potential medical use of donepezil (i.e., for memory restoration). While media headlines reference both topics, bioethics titles focus on enhancement and reflect coverage of general issues related to the CE debate. This could indicate that donepezil is rarely examined in the bioethics literature as a cognitive enhancer in its own right (i.e., for memory), but has become part of the larger debate on CE where it is taken to be an illustrative example. Alternatively, it could point to the media interpreting the primary study in different ways, i.e., as both a treatment study and an enhancement study, while bioethics articles unilaterally interpreted the study as evidence of an enhancement effect.

Our analysis of how the results of the DFSS are depicted in media and bioethics articles is consistent with the presentation of strong claims for CE that we found in headlines, titles, and colloquialisms. Amplification of a CE effect occurs both within and across individual articles as the findings of the DFSS are consistently presented at two levels (i.e., specific or extended; Additional file 3 and Additional file 4). In both literatures, the drug effect is reported with reference to three broad categories of characteristics, namely, task performance, memory, and brain, mind, or mental capacity. The way this effect is conveyed, either through improvement language (which could connote a treatment or an enhancement effect) or through explicit enhancement language, and what category of characteristic is emphasized, changes depending on the level at which the result is reported.

We found a clear trend to use explicit CE language and to report effects on memory and brain, mind, and mental capacity when articles report the findings as *extended* results. This trend is particularly salient in bioethics articles, where the majority of result claims use enhancement language at this level. The proportion of claims about DFSS results made by the media that use enhancement language does increase at the extended level; however, the majority of media claims use improvement language. When improvement language is used at this level, both literatures equally make reference to all three categories of performance improvement.

Conversely, when reporting results at the *specific* level, both literatures use a higher proportion of improvement rather than explicit enhancement language. Both literatures overwhelmingly report an improvement in task performance, followed by an improvement in memory. An improvement in brain function is only raised as a *specific* result of the study by bioethics articles. *Specific* reports of improvements in performance (and memory to a lesser degree) are often associated with discrete tasks, such as flying a flight simulator or performing an emergency procedure. However, further specification of what aspects of these broad characteristics are influenced by donepezil is rare (i.e., executive function, short or long-term memory) as is a precise description of the original level of significance of the findings or what trend contributed to this significant difference (i.e., a decrease in the performance of the pilots in the placebo group). Too few articles use enhancement language at this level to establish any trend.

Evidently, the findings of the DFSS that are reported, as well as the level of detail conveyed, change substantially as they are reported at the specific and extended levels. The direction of this change—from claims of memory and task improvement to claims of enhanced cognitive traits—as articles move away from a focused discussion of the DFSS to report generalized findings suggests that the results of this study may have been hyped as they were used to support the CE debate. However, this data also reflects the duality of study interpretations we found in the headlines, titles, and colloquialisms and may be attributable to the study being interpreted in different ways (i.e., as both a treatment and enhancement study).Our final investigation of how accurately media and bioethics articles translate and present the characteristics of the DFSS (e.g., subjects, study design) showed that overall most study characteristics are accurately identified; only a few are clearly incorrect (Figure 1). However, we found high levels of variability in claims related to the “subjects” and “methods” categories. The information provided on the ‘subjects’ category is straightforward in the DFSS, so these discrepancies stand out. There is a trend to report subject characteristics using the language of the CE debate, e.g., “healthy,” or in relation to the expectation that the drug may be useful for “fighter” or “commercial” pilots (Figure 2). Close examination of the DFSS itself shows that information on the methods used may be difficult for a lay audience to interpret, making accurate restatement of these characteristics legitimately difficult. This variable accuracy in the reporting of study characteristics, combined with our findings related to the reporting of the study’s results, suggests that the strong claims of CE effects associated with donepezil use cannot be accounted for by simple inaccuracy in reporting of the primary paper. More complex interactions seem to be governing the accurate dissemination and interpretation of the findings of the study.

We explore the potential factors involved in the hyped portrayal of the results of the DFSS and the potential ethical implications for society and clinical decision-making in the following discussion points: 1) the magnification of the connection between the results of the DFSS and the CE debate; and, 2) the ethical challenges of hyped claims of CE effects for clinicians.

### The magnification of the connection between the results of the DFSS and the CE debate

Further comparison of the results of the DFSS that were reported in media and bioethics articles with the conclusions drawn by the authors of the study in its “discussion” section provides insight into how difficult aspects of the CE debate may have contributed to how the results of the DFSS came to be reported as support for CE.

The findings presented by the DFSS in its “abstract” or “results” section (Table 3) do not claim an enhancement in performance, rather they claim that: “the donepezil group showed greater ability to retain the capacity to perform” [22] (Table 3, abstract). The majority of *specific* results reported by both media and bioethics articles may be said to have accurately conveyed this result as they claimed improvement in task performance or memory (Additional file 3 and Additional file 4).

In the discussion of the DFSS the authors move from discussing the specific effect they found to generalize an improvement in cognitive performance. They present the qualifying clause: “these results should not be interpreted to advocate widespread use of donepezil in nondemented populations” [22] before stating that “[n]onetheless” [22], their results are consistent with those of previous studies, which “have reported that cholinesterase inhibitors improve cognitive performance” [22]. As discussed above in the background section*,* the interpretation of these results as evidence that donepezil improves flight performance has since been discredited by the authors. A recent review also concluded that the DFSS only demonstrates that donepezil “*might* improve the retention of training on complex aviation tasks” [12] (emphasis added). Finally an additional study that reviewed the level of evidence provided by the DFSS and other studies on acetylcholinesterase inhibitors similarly concluded: “the available evidence does not appear to support the widely cited conclusion that donepezil improves the retention of training” [24]. Thus, while providing an acceptable lay interpretation of the data, it can be concluded that specific result claims magnify the results of the DFSS.

Beyond generalizing memory to cognition, the authors go on to dedicate the final paragraph of their discussion to raising concerns about CE, directly raising and naming the concept of CE for the first time. Careful examination of this section exposes how difficult aspects of the CE debate, namely understanding the distinction between references to CE that connote a medical term in the AD literature and those that connote CE in healthy individuals and assessing whether a study on an aging population can be considered to be a CE study, may have compounded the challenge of accurately interpreting the study, such that articles’ interpretations are understandably biased towards a CE effect.

Throughout the paragraph, the authors consider what would happen if CE becomes a possibility for intellectually intact individuals and discuss ethical issues tightly linked to the CE debate, such as access, justice, fairness, and regulation. Yet, at the same time, they imply that their study population was not healthy: they discuss the potential demand for CE from aging individuals without AD who seek to remedy their “deficits” [22]. In this way, the authors make a clear connection between the findings of their study and a CE effect, yet it remains unclear whether they intended to distinguish CE as found in the AD literature from CE discussed in the ethics debate. Thus, the *extended* result claims of CE effects that were reported in media and bioethics articles seem to be attributable, at least in part, to different messages found in the original publication as the authors attempted to connect their findings to those of other studies and, potentially, to the CE debate.

Problems created in the dissemination of research results emerging from discrepancies within one primary study are certainly not unique to the CE debate or the study we examined. For example, a study on the dissemination of attention deficit hyperactivity disorder (ADHD) research (primary studies that associate polymorphisms of a specific gene with ADHD) found: (1) internal inconsistencies between claims made in the results and those presented in the conclusions; (2) a strong conclusion claim in the summary, while data that limit this conclusion were only present in the results section; and (3) the inappropriate extrapolation of findings to therapeutic prospects [18]. These data offer support for our finding of a shift in emphasis between the data reported in the results section of the DFSS and the generalized conclusion offered in the study’s discussion that this result may lend support to the CE debate. Previous research on developments in biotechnology, including neurotechnology, has also shown that the majority of authors include both a qualifying clause in their paper that addresses the uncertainty inherent in their data, as well as a main explanation of their data, that is designed to provoke discussion with their peers. Yet, often only the main or simplified explanation is widely disseminated in print media [20,76] and observed by stakeholders exposed to media coverage of cognitive enhancement [77]. Further, explanations that were reported in the media have been found to be inconsistent with what was actually reported in the study, reflecting “different versions of future relevance” [20] as the data is interpreted and translated. This phenomenon provides greater insight into our finding that media and bioethics articles reported more generalized conclusions than qualifying clauses and helps to account for the further discrepancy observed in the facts reported by media and bioethics articles.

Finally, the presence of expectations in a field of research has also been found to influence the claims made by authors of both primary and secondary literature [20]. Brown [20] describes how anticipation of a prospective future is crucial for project development, funding, and creativity. Yet, hype around expectations can threaten the legitimacy of a research project. Unfortunately, the perpetuation of expectations rarely occurs without hype and strong claims of an effect. Where there are expectations, a spatial dynamic is often created “whereby the further we travel from the source of knowledge production, the more colourful and flamboyant become the promissory properties of knowledge” [20]. This trend is consistent with previous work on the reporting of neuroscience research [15,27]. Our current data may well indicate the presence of such a spatial dynamic. In some germinal form, we saw that the primary authors’ description of their findings moved from memory retention in the results section to CE in the discussion. This trend was magnified in claims made in media and bioethics articles given their presentation of the results of the DFSS on two levels (*specific* and *extended*). Finally, when we consider the strong claims and latent expectations for CE found in our colloquialism and headline data, there is additional support for the hypothesis that there is a distinct trend to elaborate on findings, based on expectations rather than reflecting uncertainty, as claims are further removed from the source of the data.

### The ethical challenges of hyped claims of CE effects for clinicians

One ethical implication of print media and bioethics literature hyping claims of CE effects while referencing primary research arises from the effect of hype on pre-existing expectations about CE drugs. Specifically, it has been widely recognized that one of the risks of endorsing the use of pharmaceuticals for enhancement purposes is that it may perpetuate social pressures, such as the pressure to perform, be productive, highly intelligent, or competitive [78]. Unchecked, social pressure may perpetuate social values that are not in line with the interests of the population as a whole, or do not reflect the interests of all individuals [79]. These performance-oriented values, though viewed by some as integral to a thriving society (e.g. [80]), may negatively impact individuals’ ideas of their own self-worth, fostering anxiety and discontent regarding genuinely lived experiences [78], and subsequently inducing a society that instead celebrates those traits that are the most in line with quantifiable economic and military principles of productivity. The long-term social consequences of drug use for CE purposes are unknown; however, the risk of inducing a “medicated normality”, has been raised, where a loss of cultural and social diversity, accompanied by intolerance towards difference, would manifest as an assault on the autonomy of individuals [10]. Others have raised the possibility that such drug use could induce unanticipated changes to the complex biosocial and psychological functions of the brain, altering our social behaviour in an altogether different way [81].

Research on the role of social pressure in the context of CE has isolated a “funnel phenomenon,” [82] where social pressure covertly drives individual choice in the use of methylphenidate for CE among students [82]. When prompted, stakeholders in the debate (e.g., students, health care providers) firmly believed that students’ personal values guided their decision to use methylphenidate for CE (i.e., students acted autonomously). However, they also believed that social pressure to perform was so strong in the academic community that it created “a form of social determinism leading to conformity with social values through a concession of personal values” [82]. The authors concluded that personal values are an ornamental, rather than substantial, factor in decision-making. This finding has substantial implications in the context of our own results. If health care professionals believe individuals are making autonomous choices, yet external pressures are implicitly shaping these decisions, the task of determining whether an individual is being coerced to change a trait becomes challenging and worthy of attention from an ethics standpoint.

It has been recognized that as requests for “neuroenhancements” enter the clinical realm, physicians will be left with the difficult task of interpreting strong claims, modifying expectations, and addressing social pressure. However, recently published guidelines on how neurologists should deal with these requests [8] concluded that neurologists were sufficiently aware of these social factors and thus left out specific recommendations on how to take social pressures into account, opting instead to support a physician-patient discussion [8]. As demonstrated by the previous data, there is a risk that within the context of the patient-physician relationship it will be difficult to determine whether the individual is in fact making informed decisions based on appropriate claims. Further, in its absence from the guidelines, the presumed importance of this issue may be overlooked. An extended patient-physician discussion may uncover reasons for the request; however, without specific guidelines to address misguided expectations and related social pressure in the clinical context, physicians may consider further attention to these issues unnecessary or even consider the presence of social pressure as an ethically justifiable reason to prescribe.

It has been suggested that the description of the lack of data on the safety and efficacy of cognitive enhancers would deter physicians from prescribing them [8]. However, as our study shows, this dearth of scientific evidence can be difficult to grapple with due to hyping of the results in media and bioethics literature. Indeed, these very guidelines cited the DFSS as evidence in support of the efficacy of these drugs [8]. Clearly, there is an important interaction between strong public claims about CE, pre-existing social pressure, the existence of expectations for CE, and the ability to adequately disseminate information on safety and efficacy. Mitigating misguided claims, social pressure, and expectations will be an ongoing process. Organizations have begun developing explicit recommendations on how to deal with social pressures surrounding CE agents [10]; however, more work is needed to help physicians integrate knowledge of social factors and circumstances that shape decision-making [83]. As the CE debate continues, caution should be further applied to avoid complicity with negative social norms that surround intellectual disability. Attention to disability ethics literature may help bioethicists and clinicians become acquainted with the obligations we have to support people with disabilities.

Another area to explore is how information could be provided to the public based not on single studies, but on more comprehensive and authoritative reviews of the literature. Primary researchers should consider means to reduce misappropriation of their work, and journalists and editors should be accountable for writing headlines that draw in readers while conserving accuracy.

### Limitations

In spite of broad searches and the use of multiple databases, our sample may not be exhaustive of all articles on non-medical use of donepezil with reference to the DFSS. The small sample size, which may well reflect the scope of the CE debate, makes it difficult to make any wide-reaching conclusions. Like other qualitative content and discourses analyses, though coding was double-checked and thoroughly pilot tested, controlling for subjectivity of data gathering and analysis is challenging and represents another study limitation. Further, our findings should not be interpreted as an accusation of the authors of the DFSS or of the specific journalists or scholars who were authors of the media and bioethics articles in our sample. We understand that the reported media statements reflect an amalgamation of data and opinions and do not necessarily reflect the opinions of the authors. Accordingly, the content of our sample should be viewed as a reflection of what members of the public have access to, rather than the individual voice of the author *per se,* and our findings should be considered a detailed exploration of the challenges of interpreting complex data and their contribution to contemporary ethics debates about neurological advances in healthcare and beyond.

## Conclusion

Our findings regarding which factors contribute to the presence of strong media and bioethics claims that donepezil has a CE effect support the general finding that both media and academic literature often magnify the limited conclusions that can be drawn from basic research, putting them in line with expectations that may be heavily influenced by prominent social pressures. A complex interaction between the authors of primary and secondary literature, generated in part by the tenuous distinction between treatment and enhancement that sets the premise for the CE debate, and in part by the presence of widespread expectations and social pressures, may contribute to this phenomenon. Caution is needed to both explicitly account for social pressure and acknowledge the limited data on safety and efficacy as we continue to discuss the use of drugs for CE, particularly in the clinical context.

## Endnotes

^a^We consider the term “CE drug” as well as the practice descriptor “CE” to be problematic. They imply that increasing cognition is both possible and beneficial. We further describe this problem throughout the manuscript. However, to enhance readability and be consistent with the current literature, we use these terms to reference those drugs and related practices that have been discussed as being capable of increasing cognition.

^b^Though we recognize the transient definition of medical need, for the purpose of this article we employ Daniels’ definition “[c]haracterising medical need…implies a contrast between medical services that treat disease (or disability) conditions and uses that merely enhance human performance” [17] as it reflects what is commonly held to be the distinction between drug use for medical need and that for enhancement purposes.

^c^For the purpose of this paper we will refer to this study as the Donepezil Flight Simulator Study (DFSS; we encourage readers unfamiliar with this study to consult Table 1 for further details).

## Abbreviations

(DFSS): The Yesavage *et al*. 2002 study is referred to as the “Donepezil flight simulator study; M: International print media articles as; BE: Interdisciplinary bioethics articles as; CE: Cognitive enhancement; ADHD: Attention deficit hyperactivity disorder as.

## Competing interests

The authors declare that they have no competing interests.

## Authors’ contributions

LW led the development of the study design, data gathering and analysis, and writing of the paper. CF and ER provided input on the study design, coding strategy and reliability, and the manuscript. All authors read and approved the final manuscript.

## Pre-publication history

The pre-publication history for this paper can be accessed here:

http://www.biomedcentral.com/1472-6939/15/37/prepub

## Supplementary Material

Additional file 1 **Print media sample. **List of all media articles in the sample. Click here for file

Additional file 2 **Interdisciplinary bioethics literature sample.** List of all bioethics articles in the sample. Click here for file

Additional file 3 **Statements of the results of the DFSS as reported in media articles.** Table reporting statements found in media articles. Click here for file

Additional file 4 **Statements of the results of the DFSS as reported in bioethics literature. **Table reporting statements found in bioethics articles. Click here for file
