# Supplementary material for: Generating genius: how an Alzheimer’s drug became considered a ‘cognitive enhancer’ for healthy individuals
Source: BMC Med Ethics. 2014 May 12;15:37. doi: 10.1186/1472-6939-15-37 (PMC4063424; doi:10.1186/1472-6939-15-37)
Supplement: Additional file 1 — Print media sample. List of all media articles in the sample. [file 1472-6939-15-37-S1.docx]

Appendix 1: Print media sample.

1. **Briefs: New clues in Alzheimer's**. In: *The Record*. 2002 July 29.

2. **Drug found to improve performance among pilots**. In: *Airline Industry Information*. 2002 July 9.

3. **New memory pill- in brief**. In: *The Times*. 2002 July 9.

4. **New potential for Alzheimer's and cholesterolemia markets**. In: *MedMark Media*. 2002 August 1: 22.

5. **A stimulating debate**. In: *Irish Independent*. 2009 July 7.

6. Arnst C. **"I can't remember"; drugs to stave off age-induced memory impairment may be on the horizon**. In: *Business Week*. 2003 September 1.

7. Balfour N. **Superbrain future, recycled water, better oxygen supply, Alzheimer drug boost for healthy memory**. In: *The Sunday Times*. 2002 August 11.

8. Bee P. **Smart drugs for straight A's**. In: *The Times*. 2007 May 14.

9. Begley S. **New ethical minefield: Drugs to boost memory and sharpen attention**. In: *The Wall Street Journal*. 2004 October 1.

10. Burne J. **Can taking a pill really make you brainy?** In: *Daily Mail*. 2007 December 26.

11. Dillon W. **A clever idea? The drugs that make you smart**. In: *Irish Independent*. 2005 February 2.

12. Evenson B. **Alzheimer's drug improves 'working memory': Side effects possible: Older pilots remember training better in test**. In: *National Post*. 2002 July 9.

13. Gibson E. **Mental pick-me-ups: The coming boom**. In: *Business Week*. 2008 December 29.

14. Healy M. **Newest drugs act as brain's fountain of youth**. In: *The Grand Rapids Press*. 2005 January 18.

15. Healy M. **Sharper minds**. In: *Los Angeles Times*. 2004 December 20.

16. Jaffe S. **Scientists test theories on aging and their resolve: Researchers become case studies for their own work**. In: *The Plain Dealer*. 2002 December 16.

17. Kher U. **Can you find concentration in a bottle?** In: *Time (Canadian Edition)*. 2006 January 16: 98-99.

18. Laurance J. **Messing with our minds**. In: *The Independent*. 2005 January 18.

19. Laurance J. **Mind-enhancing drugs: Are they a no-brainer?** In: *The Independent*. 2009 June 19.

20. Macintyre B. **Extreme makeover for the brain**. In: *The Times*. 2004 November 27.

21. McCook A. **Alzheimer's drug ups memory in healthy pilots**. In: *Reuters Health E-Line*. 2002 July 8.

22. Miller H, Longtin D. **Smart pill, anyone?** In: *The Washington Times*. 2004 July 21.

23. Morgan J. **Brain-boosting drugs could soon become the smart choice**. In: *The Herald*. 2008 January 3.

24. O'Neil J. **Testing medicine and memory**. In: *The New York Times*. 2002 July 9.

25. Reucroft S, Swain J. **Smart drug**. In: *The Boston Globe*. 2002 July 16.

26. Rosenthal R. **Data needed on cognition-enhancing drugs**. In: *Pediatric News*. 2009 August 1.

27. Stuttaford T. **Memory drug has landed-medical briefing**. In: *The Times*. 2002 July 11.
