# Supplementary material for: Generating genius: how an Alzheimer’s drug became considered a ‘cognitive enhancer’ for healthy individuals
Source: BMC Med Ethics. 2014 May 12;15:37. doi: 10.1186/1472-6939-15-37 (PMC4063424; doi:10.1186/1472-6939-15-37)
Supplement: Additional file 2 — Interdisciplinary bioethics literature sample. List of all bioethics articles in the sample. [file 1472-6939-15-37-S2.docx]

Appendix 2: Interdisciplinary bioethics literature sample.

1. Bolt LL: **True to oneself? Broad and narrow ideas on authenticity in the enhancement debate**. *Theor Med Bioeth* 2007,**28**:285-300.

2. Bush SS: **Neurocognitive enhancement: Ethical considerations for an emerging subspecialty**. *Appl Neuropsychol* 2006,**13**:125-136.

3. Chatterjee A: **Cosmetic neurology and cosmetic surgery: Parallels, predictions, and challenges**. *Camb Q Healthc Ethics* 2007,**16**:129-137.

4. Chatterjee A: **Cosmetic neurology: The controversy over enhancing movement, mentation, and mood**. *Neurology* 2004,**63**:968-974.

5. Chatterjee A: **The promise and predicament of cosmetic neurology**. *J Med Ethics* 2006,**32**:110-113.

6. Cheshire W: **The pharmacologically enhanced physician**. *Virtual Mentor* 2008,**10**:594-598.

7. Choudhury S, Nagel SK, Slaby J: **Critical neuroscience: Linking neuroscience and society through critical practice**. *Biosocieties* 2009,**4**:61-77.

8. de Jongh R, Bolt I, Schermer M, Olivier B: **Botox for the brain: Enhancement of cognition, mood and pro-social behavior and blunting of unwanted memories**. *Neurosci Biobehav Rev* 2008,**32**:760-776.

9. Douglas T: **Moral enhancement**. *J Appl Philos* 2008,**25**:228-245.

10. Farah MJ, Illes J, Cook-Deegan R, Gardner H, Kandel E, King P, Parens E, Sahakian B, Wolpe PR: **Neurocognitive enhancement: What can we do and what should we do?** *Nat Rev Neurosci* 2004,**5**:421-425.

11. Forlini C: **Examining discourses on the ethics and public understanding of cognitive enhancement with methylphenidate***.* Montreal: Université de Montréal; 2008.

12. Gerlai R: **Memory enhancement: The progress and our fears**. *Genes Brain Behav* 2003,**2**:187-190.

13. Lanni C, Lenzken SC, Pascale A, Del Vecchio I, Racchi M, Pistoia F, Govoni S: **Cognition enhancers between treating and doping the mind**. *Pharmacol Res* 2008,**57**:196-213.

14. Larriviere D, Williams MA, Rizzo M, Bonnie RJ: **Responding to requests from adult patients for neuroenhancements: Guidance of the ethics, law and humanities committee**. *Neurology* 2009,**73**:1406-1412.

15. Mehlman MJ: **Cognition-enhancing drugs**. *Milbank Q* 2004,**82**:483-506.

16. Mehlman MJ, Berg JW: **Human subjects protections in biomedical enhancement research: Assessing risk and benefit and obtaining informed consent**. *J Law Med Ethics* 2008,**36**:546-549.

17. Normann C, Berger M: **Neuroenhancement: Status quo and perspectives**. *Eur Arch Psychiatry Clin Neurosci* 2008,**258 Suppl 5**:110-114.

18. Racine E, Illes J: **Neuroethical responsibilities**. *Can J Neurol Sci* 2006,**33**:269-277, 260-268.

19. Riis J, Simmons JP, Goodwin GP: **Preferences for enhancement pharmaceuticals: The reluctance to enhance fundamental traits**. *J Consum Res* 2008,**35**:495-508.

20. Vincent JA: **Science and imagery in the 'war on old age'**. *Ageing Soc* 2007,**27**:941-961.

21. Warren OJ, Leff DR, Athanasiou T, Kennard C, Darzi A: **The neurocognitive enhancement of surgeons: An ethical perspective**. *J Surg Res* 2009,**152**:167-172.

22. Whitehouse PJ: **Paying attention to acetylcholine: The key to wisdom and quality of life?** *Prog Brain Res* 2004,**145**:311-317.
