# Supplementary material for: Generating genius: how an Alzheimer’s drug became considered a ‘cognitive enhancer’ for healthy individuals
Source: BMC Med Ethics. 2014 May 12;15:37. doi: 10.1186/1472-6939-15-37 (PMC4063424; doi:10.1186/1472-6939-15-37)
Supplement: Additional file 3 — Statements of the results of the DFSS as reported in media articles. Table reporting statements found in media articles. [file 1472-6939-15-37-S3.docx]

Additional file 3: Statements of the results of the DFSS as reported in media articles.*

|  | **Task Performance** | **Memory** | **Brain, Mind, Mental Capacity** |
| --- | --- | --- | --- |
| *Enhancement* | --- | *“has turned out to enhance memory and concentration in healthy people”* [[49](#_ENREF_49)]  *“could lead to a memory pill”*[[53](#_ENREF_53)]  *“the success of the pilots' study does demonstrate that memory-enhancing pills are possible”* [[28](#_ENREF_28)]  *“Even memory enhancement seems within reach, at least for older folks.”* [[55](#_ENREF_55)] | “was shown… to enhance mental performance” [[51](#_ENREF_51)]  *“It is one of three prescription medications… that have been shown to enhance certain mental powers. The other two are… and donepezil.”* [[35](#_ENREF_35), [36](#_ENREF_36)]  *“drugs investigated for their mind-enhancing properties include donepezil”* [[56](#_ENREF_56)] |
| *Significance* | “performed significantly better” [[48](#_ENREF_48)]❶  “reportedly performed significantly better” [[57](#_ENREF_57)]❶  “performed significantly better” [[32](#_ENREF_32)] | “were significantly better at recalling” [[46](#_ENREF_46)]❶❺ | *“Studies have shown that these drugs can produce significant mental gains in normal, healthy subjects.”* [[35](#_ENREF_35), [36](#_ENREF_36)] |
| *Nonspecific* | “was successfully tested” [[53](#_ENREF_53)] | --- | *“The era of "brain-doping" may be looming.”* [[49](#_ENREF_49)] |
| *Specific* | “Overall, pilots who took the drug showed little difference in performance ... The performance of those who took a placebo declined” [[*48*](#_ENREF_48)*]* | --- | --- |
| *Improvement + Superlative* | “were measurably better able to perform” [[28](#_ENREF_28)]❶❷  “especially excelled” [[50](#_ENREF_50)]❸❹  “did markedly better” [[58](#_ENREF_58)]❶❸❺  “performed markedly better” [[59](#_ENREF_59)]❸  “There was a marked difference between how the two groups dealt with ❸ situations” ([[52](#_ENREF_52)]  “There was a marked difference between the groups when dealing with ❸” [[60](#_ENREF_60)] | “recalled ❶❺ notably better” [[55](#_ENREF_55)]  “Ritalin-type drugs… clearly improve attention and memory… so does donepezil” [[51](#_ENREF_51)]❶ | *---* |
| *Improvement or “Bettering”* | “improved performance” [[49](#_ENREF_49)]❷  “improves a pilot's performance and skills” [[32](#_ENREF_32)]❸❹  “can improve the performance” [[57](#_ENREF_57)]  “showed improved… and performance” [[61](#_ENREF_61)]❶❷  “has been shown to boost the performance” [[62](#_ENREF_62)]❶  “has been shown to boost the performance” [[56](#_ENREF_56)]❶❸  “helped ❹ better and handle ❶❸” [[63](#_ENREF_63)]  “showed Improved performance” [[64](#_ENREF_64)]❶  “performed better” [48]; [[60](#_ENREF_60)]; [[52](#_ENREF_52)]❶  “performed better” [[50](#_ENREF_50)]❶  “performed better”[[56](#_ENREF_56)]❶❸  “did better” [[65](#_ENREF_65)]❶❸  “did better” [[66](#_ENREF_66)]❸❹  “were more adept (at extremely)”[[64](#_ENREF_64)]❶❷  “coped better with the flood of information” [[49](#_ENREF_49)] | “showed improved memory…” [[61](#_ENREF_61)]❶❷  “improved the memory of fighter pilots” [[33](#_ENREF_33)]❺  “could improve the memory of fighter pilots” [[31](#_ENREF_31)]❶❺  “improves long-term and recent memory and recognition tasks” [[61](#_ENREF_61)]  *“Thought to boost memory”* [[33](#_ENREF_33)]  *“boost for healthy memory”* [[49](#_ENREF_49)]  *“There is already evidence that even nimble memories can be improved.”* [[28](#_ENREF_28)] | *“has also been shown to boost the brain function”* [[67](#_ENREF_67)]  *“also has been found to boost the brain function”* [[35](#_ENREF_35), [36](#_ENREF_36)]  *“But Modafinil does not stand alone in its ability to sharpen the mind… donepezil has been shown to… boost performance in tests of cognitive skill”* [[67](#_ENREF_67)]  *“[these drugs] also have the potential to deliver unexpected psychological benefits to the rest of the population”* [[52](#_ENREF_52)] |
| *Improvement + Dimunitive* | *“may give a boost”* [[47](#_ENREF_47)]  *“may boost highly skilled performance, where concentration and alertness are prerequisites”* [[60](#_ENREF_60)]  *“may also boost performance in situations where concentration and alertness are vital”*[[52](#_ENREF_52)]  *“might increase alertness and concentration to minimize risk of pilot error and maximize endurance”* [[56](#_ENREF_56)], [[62](#_ENREF_62)]  *“Both drugs are thought to boost highly skilled performance, where concentration and alertness are prerequisites”* [[56](#_ENREF_56)]  “performed slightly better” [[50](#_ENREF_50)]❶ | “showed some improvement in short-term memory” [[47](#_ENREF_47)]  “slightly helped airline pilots retain” [[66](#_ENREF_66)]❷❺  *“may offer more powerful, better targeted and longer lasting improvements in mental acuity”* [[60](#_ENREF_60)]  *“may well improve memory”* [[46](#_ENREF_46)]  *“may offer help to older people with benign memory loss”* [[32](#_ENREF_32)]  “When confronted with ❸ new transponder codes and a series of air traffic control commands, older pilots who took donepezil… were less likely to forget ❺” [[48](#_ENREF_48)] | *“Drugs already on the market… have been shown in small studies to improve the performance of healthy brains, though not by much.”* [[51](#_ENREF_51)] |
| All report a change in general performance. Some specify changes in: ❶flight simulator; ❷complex task; ❸emergency; ❹landing; or, ❺training or learning. Those highlighted in red are results of the DFSS that have been reported at the “extended” level, while those in black are findings reported at the “specific” level. Statements shown have been truncated. The most common roots are: “Donepezil _________”; or, “Pilots who took donepezil________ than the control group”. If the root is different, more context is given.  * Reported findings are divided by effect on performance, memory, and brain, mind or mental capacity, and are further divided by how those effects are presented. The results presented in M and B were also divided into two levels, specific results and extended results (italicized). | | | |
