# Supplementary material for: Generating genius: how an Alzheimer’s drug became considered a ‘cognitive enhancer’ for healthy individuals
Source: BMC Med Ethics. 2014 May 12;15:37. doi: 10.1186/1472-6939-15-37 (PMC4063424; doi:10.1186/1472-6939-15-37)
Supplement: Additional file 4 — Statements of the results of the DFSS as reported in bioethics literature. Table reporting statements found in bioethics articles. [file 1472-6939-15-37-S4.docx]

Additional file 4: Statements of the results of the DFSS as reported in bioethics literature.*

|  | **Task Performance** | **Memory** | **Brain, Mind, Mental Capacity** |
| --- | --- | --- | --- |
| *Enhancement* | “did enhance performance ” [[40](#_ENREF_40)]  “the two areas of enhanced performance were ❹, and handling ❸, both of which are attention-intensive tasks” [[38](#_ENREF_38)] | *“evidence for enhanced memory encoding and increased retention in normal humans exists”* [[68](#_ENREF_68)]  *“Research on drugs that may enhance…the retention of complex skills…suggest that the possibilities for biomedical enhancement are likely to grow rapidly in coming years”* [[69](#_ENREF_69)]  *“the scepticism exhibited by some who argue finding such drugs [`smart drugs` i.e., memory enhancing agents] is impossible is perhaps not warranted”* [[45](#_ENREF_45)] | *“Research has also indicated possible venues for cognitive enhancement”* [[70](#_ENREF_70)]  *“could possibly become enhancers of cognitive performance”* [[39](#_ENREF_39)]  *“There is associated research into brain-enhancement drugs…which have enhanced the cognitive performance of aircraft pilots”* [[42](#_ENREF_42)]  *“Prescription medications such as donepezil…are being investigated for their potential to enhance memory, cognition, and executive function in healthy individuals”* [[6](#_ENREF_6)] |
| *Significance* | “showed a highly significant increase in performance” [[71](#_ENREF_71)]❷ | “had significantly better post-training retention” [[6](#_ENREF_6)]❷❸ | --- |
| *Nonspecific* | *“one intriguing report suggests an effect in the setting of highly skilled performance”* [[72](#_ENREF_72)]  “has shown efficacy“ [[45](#_ENREF_45)] | *“Attention, memory, and learning can also be modulated in healthy people”* [[43](#_ENREF_43)] | --- |
| *Specific* | “In the donepezil group, the flight performance on day 30 was found to be similar to performance after initial training, whereas in the placebo group, flight performance declined.” [[24](#_ENREF_24)]** | --- | --- |
| *Improvement + Superlative* | --- | --- | --- |
| *Improvement or “Bettering”* | “has been found to improve the performance” [[73](#_ENREF_73)]❶❷  “performed better” [[72](#_ENREF_72)]❶❷❸  “performed better” [[74](#_ENREF_74)]❶❸  “improve normal performance in laboratory vigilance tests” [[43](#_ENREF_43)]  “improves the performance of commercial airline pilots” [[39](#_ENREF_39)]❶  “improves performance” [[75](#_ENREF_75)]  “improved the subjects’ ability to respond to ❶, in-flight ❸” [[54](#_ENREF_54)]  “outperformed a control group on tests of performance” [[44](#_ENREF_44)]  “appeared to improve their performance when compared to those on placebo” [[38](#_ENREF_38)] | “it improved retention of ❺and episodic (long-term) memory” [[40](#_ENREF_40)]  “was shown to improve retention” [[76](#_ENREF_76)]]❷❺  “were able to better retain information” [[70](#_ENREF_70)]  *“can improve memory and executive function”* [[8](#_ENREF_8)]  *“these drugs may not simply…improve recall. Some of them may also improve executive function”* [[75](#_ENREF_75)] | “has improved cognitive performance” [[41](#_ENREF_41)]  *“improve cognition”* [[75](#_ENREF_75)]  *“there are several drugs that can improve cognitive abilities such as intelligence, concentration, learning, and memory”*[[44](#_ENREF_44)] |
| *Improvement + Dimunitive* | *“may improve normal performance under some circumstances”* [[74](#_ENREF_74)] | --- | --- |
| All report a change in general performance. Some specify changes in: ❶ flight simulator; ❷complex task ; ❸emergency ; ❹ landing; or, ❺ training or learning. Those highlighted in red are results of the DFSS that have been reported at the “extended” level, while those in black are findings reported at the “specific” level. Statements shown have been truncated. The most common roots are: “Donepezil _________”; or, “Pilots who took donepezil________ than the control group”. If the root is different, more context is given.  *Reported findings are divided by effect on performance, memory, and brain, mind or mental capacity, and are further divided by how those effects are presented. The results presented in M and B were also divided into two levels, specific results and extended results (italicized).  **Of note, [[24](#_ENREF_24)] was the only bioethics article that provided a critique of the DFSS. | | | |
